# Perceptions of Health and Safety among Immigrant Latino/a Dairy Workers in the U.S.

**DOI:** 10.3389/fpubh.2016.00106

**Published:** 2016-05-30

**Authors:** Lauren M. Menger, Florencia Pezzutti, Teresa Tellechea, Lorann Stallones, John Rosecrance, Ivette Noami Roman-Muniz

**Affiliations:** ^1^Department of Psychology, College of Natural Sciences, Colorado State University, Fort Collins, CO, USA; ^2^Department of Anthropology, College of Liberal Arts, Colorado State University, Fort Collins, CO, USA; ^3^Department of Environmental and Radiological Health Sciences, College of Veterinary Medicine and Biomedical Sciences, Colorado State University, Fort Collins, CO, USA; ^4^Department of Animal Sciences, College of Agricultural Sciences, Colorado State University, Fort Collins, CO, USA

**Keywords:** dairy industry, immigrant workers, Latino/a, safety, focus groups

## Abstract

The U.S. dairy industry is increasingly relying on an immigrant workforce to help meet growing demands. Due to scant research, little is known about the factors related to workplace safety among this occupational group. The purpose of this study was to identify dairy worker perceptions of the barriers to and facilitators for enhancing workplace safety. Focus groups (FG) were conducted with 44 immigrant Latino/a workers from 2 dairies in South Dakota and 1 dairy in Colorado to gain firsthand insights into their work experiences. Interviews were conducted in Spanish, audio recorded, transcribed, and translated into English. Results were analyzed through a two-step qualitative coding process. The Contributing Factors in Accident Causation model was used as a guiding framework. Promising points of intervention identified were related to the workers, the work itself, the physical environment, equipment issues, the social–psychological environment, and management/organizational factors. Suggestions for how to improve safety outcomes in the dairy industry are provided. It is likely that the dairy industry will continue to employ a growing number of immigrant workers. Therefore, these findings have significant implications that can be used to guide the development of culturally congruent policies and practices.

## Introduction

The U.S. dairy industry ranks second among major world producers, supplying 14.6% of the world’s milk supply (1). Since the introduction of new milking technologies, the industry has shifted toward a high efficiency model with increasing herd sizes (2). With the trend toward larger herds has come a growing reliance on an immigrant, primarily Latino/a, workforce (3). The federal government defines Latino/a (used interchangeably with Hispanic or of “Spanish origin”) as “a person of Cuban, Mexican, Puerto Rican, South or Central American, or other Spanish culture or origin regardless of race” (4). Estimates of immigrant Latino/a workers on U.S. dairies have been reported as high as 94% (5).

Latinos/as tend to share a common set of values that are distinct from those found in mainstream American culture, including higher levels of in-group collectivism and familism (importance of family), stronger adherence to traditional gender roles, and greater acceptance of hierarchical power structures (6). It is difficult to generalize these commonalities given the great diversity across the Latino/a population in terms of country of origin, parental ethnicity (or ethnicities), length of time in the U.S., and levels of acculturation and language fluency (6). Clearly, managing a culturally diverse, primarily immigrant workforce poses unique challenges to dairy industry leaders when it comes to improving health and safety (7).

The demands of the dairy industry on worker health are many. On a daily basis, dairy workers are faced with diverse challenges, including high workload and time pressures, equipment failures and technological difficulties, and hazardous working conditions (8). As a result, the dairy industry has long been recognized as a high-risk occupation (9–12), characterized by elevated rates of injury, illness, and turnover (13). In fact, it is one of the few industries that experienced an increase in non-fatal injuries between 2010 and 2011 (14). Some of the more common occupational hazards include risks associated with machinery operation and repair, large animal handling, respiratory exposures, ergonomic risks including repetitive motions and high muscle forces required in parlor milking, and fatigue due to long hours and physical demands (2, 15–17). Although dairy operations employing more than 10 workers are subjected to regulations by the U.S. Department of Labor’s Occupational Safety and Health Administration (OSHA), and currently, there are Local Emphasis Programs (LEP) targeting the dairy industry in several states as a result of work-related risks and fatalities; at this time, there is no federally mandated occupational health and safety training in the dairy industry (18). Advocacy groups, such as the Worker Justice Center of New York^1^ and the United Farm Workers of America,^2^ have highlighted the occupational health and safety of dairy workers as key issues due to recent incidents on dairy farms in several states.

Data regarding the incidence and prevalence of occupational injuries and illnesses among immigrant Latino/a dairy workers are scarce due to limitations in reporting systems and immigrant workers’ reluctance to report injuries or illnesses due to fear of negative employment consequences (18). However, various factors suggest immigrant Latino/a workers may be at increased risk of work-related injury and illness. Many immigrant dairy workers are young, inexperienced, have limited education, know little to no English, are likely unaware of the harms of working on a dairy, and may not have developed the skills needed for learning job tasks and safety procedures. Smith-Jackson et al. (19) surveyed agricultural workers and found that Latino/a workers had lower safety self-efficacy compared with their Anglo-American counterparts. Many Latino/a workers also share a general health belief that injury and illness is outside of individual control, influencing their acceptance of occupational safety policies and procedures as well as their receptivity to safety training programs (20).

Immigrant workers also face a number of psychosocial conditions that are different from domestic workers, such as working and living in a foreign country away from family and friends and social isolation due to cultural and linguistic barriers (8). These circumstances may make immigrant workers more prone to depression, anxiety, substance abuse, and even suicide (21, 22). Due to these psychosocial conditions and the aforementioned daily challenges faced by dairy workers, they are also subjected to high levels of work stress. In fact, farming has been listed as 1 of the 10 most stressful occupations worldwide (23). These psychosocial strains could influence productivity and performance in addition to safety outcomes.

The promotion of health and safety are high priorities for dairy industry leaders, yet there has been little research exploring immigrant worker perceptions regarding the determining factors of these outcomes (13). With the growing prevalence of Latino/a workers in the U.S. dairy industry, and the U.S. workforce more generally, more research is needed to help organizations develop culturally congruent policies, practices, and programs in accordance with the job-related attitudes, values, and behaviors of Latino/a workers. This study conducted focus groups (FG) to better understand dairy worker perceptions of the barriers to and opportunities for enhanced safety, with the goal of developing culturally appropriate job and safety training programs for this underserved and vulnerable working population.

This study adopted a systems approach by attempting to shed light on the environmental, organizational, individual, and relational factors that influence dairy worker safety and productivity outcomes. Specifically, the Contributing Factors in Accident Causation model (24, 25) was used to guide the data collection and analysis. The Contributing Factors in Accident Causation model is a comprehensive model acknowledging the influential role of management, workers and coworkers, and the social–psychological environment in addition to the classic human factors variables, including the physical environment (i.e., influences in the environment), equipment (i.e., tools or machinery in the work environment), and the nature of the work tasks (i.e., design of the work itself). Shaw and Sanders (25) define management as all procedures, practices, and policies implemented by all levels of management across the organization. The workers and coworkers refer to the individual level physical and psychological limitations that contribute to the occurrence of accidents. Finally, the social/psychological environment refers to the social climate within the organization.

The overall goal of this study was to identify ways to develop more culturally congruent human resource policies, procedures, and practices tailored to immigrant Latino/a dairy workers in order to enhance safety in the dairy industry. Culturally congruent approaches are focused on adapting to the characteristics and needs of the culture that they are aiming to influence (26). As stated by Schenker and Gunderson (3), “with immigrants representing the majority of dairy workers, understanding the causes of illness and injury need to take into account the different perceptions, understanding, and behaviors that may be associated with being an immigrant … efforts to prevent injury and illness, or to treat those outcomes when they do occur, need to be sensitive to the realities of the immigrant worker” (pp. 185–186).

## Materials and Methods

### Procedures

A convenience sample of three dairies, one in Colorado and two in South Dakota, with which the research team had previously exiting contacts were recruited. Upon agreeing to participate, dairy owners were asked to announce the opportunity and encourage workers to participate. Recruitment flyers were posted in both English and Spanish. Only Latino/a dairy workers were eligible for participation.

Using the process of focus groups data collection followed Krueger (27), 45–95 min (M = 63.57, SD = 19.45) focus groups were conducted before or after work shifts on-site in a private room. Participants were asked to describe their previous work experience as well as their current job on the dairy, including tasks, responsibilities, and productivity influences as well as job training received. They were also asked about the quality and nature of communication with their manager/s and coworkers [e.g., “*How would you describe your experience communicating with your manager(s)?*”], with a focus on the influence of language and culture. The remainder of the interviews focused on safety (e.g., “*What does working safely around the dairy mean to you?*”), including perceived importance, organizational policies and procedures, and safety training. Interviews were conducted using a structured interview guide, but with flexibility to allow for the emergence of other topics perceived as important to the workers. Demographic information was not formally collected in order to make participants feel more comfortable being honest and open in their comments; however, some information (e.g., country of origin, time in dairy industry) was collected during the group discussions. Confidentiality was assured, and written consent was obtained from all participants before starting the focus groups. It was explained and emphasized to participants that their identity and input would be protected, no names or information would be collected that could breach confidentiality and anonymity, and managers and owners would not have access to who had said what. They were also assured that transcriptions and tapes would be kept at the University office in a locked file cabinet and that any written summaries, reports, or publications would only contain aggregate data and would not include the names of their respective dairies.

A Spanish bilingual–bicultural medical anthropologist conducted participant observation in the dairies, which involved living, working, and spending time with the workers in order to better understand their point of view. Participant observation was used to identify key activities and possible questions for the focus groups and to gain understanding of the workers’ realities in their work place as well as outside with their families and peers in the trailers where they lived. This led to establishing rapport and building trust with workers, which was key to having more open conversations during focus groups. The medical anthropologist lived in the farm trailers and participated in all shifts and work activities on the dairy farm. These tasks included herding, feeding, milking and palpating the cows, helping veterinarians in the artificial insemination process, delivering calves, following protocols for after deliveries, working in the pastures, repairing irrigation systems, transportation and storage of cattle feed, checking the milk tanks, cleaning and helping with maintenance, and spending time after work at trailer gatherings, parties, lunches, etc. The participants integrated their knowledge in their role as co-researchers instead of mere subjects of the study.

All focus groups were conducted in Spanish and recorded. Audio recordings were translated into English and transcribed by a bilingual research assistant. Participants were compensated with a $35. All materials and procedures were approved by the Colorado State University Institutional Review Board before the initiation of the study.

### Data Analysis

Data analysis was conducted in two stages. First, two members of the research team independently completed open coding of each transcript and met to generate an initial list of themes. Discrepant opinions were discussed until consensus was achieved. For instance, if one member of the research team thought a participant was referring to an existing safety procedure and the other member of the research team thought the participant was making a suggestion for a new safety procedure, they would reread the text together and discuss until agreement was achieved. Each theme was operationally defined and, when necessary, assigned example quotes from the transcripts to demonstrate the nature of the category for all coders. This initial list of themes was then fitted to the Contributing Factors in Accident Causation model (24), a comprehensive model acknowledging the influential role of management, the individual workers/coworkers and the social–psychological environment in addition to the classic human factors variables, including the physical environment, equipment, and the nature of the work itself. A Latino/a member of the research team with extensive experience training Latino/a dairy workers in the U.S. read all transcripts and audited the process. Two other members of the research team (an epidemiologist and an ergonomist) also audited the analysis process. One member of the research team then applied the final themes to all transcripts. A second member of the research team reviewed 25% of the coding for each of the seven transcripts to ensure appropriate application of the codes, resulting in 96% agreement.

## Results

A total of 44 dairy workers were interviewed during 2 focus groups at 1 South Dakota dairy (*N* = 6, *N* = 6), 2 at another South Dakota dairy (*N* = 7, *N* = 7), and 3 at a Colorado dairy (*N* = 5, *N* = 6, *N* = 7). Four participants from the Colorado dairy were female, and the rest of the participants across all focus groups were male. Approximately half of the participants were from Mexico, and the rest were from Central America (primarily Guatemala), Peru, and Puerto Rico. Themes reflect common feelings across participants and are presented in line with the Contributing Factors in Accident Causation model categories as follows: worker/coworker, work itself, physical environment, equipment, social–psychological environment, and management/organizational factors. When quoting participants, words in [brackets] were changed to protect the anonymity of the workers.

### Worker/Coworker

Results at the individual level of the workers/coworkers fell under three main themes – occupational history/dairy experience, job-related knowledge, and work ethic/motivation.

#### Occupational History/Dairy Experience

Participants came from diverse occupational backgrounds and had been working at their respective dairies for anywhere between 2 and 26 years (M = 3.76 years, SD = 5.43 years, *N* = 43). Fourteen workers reported having work experience at other dairies between 2 months and 7 years (M = 2.37, SD = 2.20), and a handful of workers had experience milking cows by hand in their home country. Participants had an array of previous work experiences. The most often mentioned previous jobs were in construction, the meat industry (e.g., poultry processing plant, cattle ranch), various factories (camera, auto, boat, agrochemical products, plastics, food products, hot air balloons), and restaurants. Other previous jobs included blacksmith, businessman, housekeeper, florist, mall security, and mining. Some workers perceived their job in the dairy industry as safer compared to other jobs, such as working in a slaughterhouse.

Participants were asked to describe their reasons for selecting their current job. Some said they chose their job out of necessity, and others mentioned desirable aspects of the job, such as stability (often comparing the year-round work in the dairy industry to temporary/seasonal work in other industries), good pay compared to other industries/dairies, benefits (e.g., housing, medical benefits), and a preference for working with cows/animals. Some workers reported finding the job through a relative or friend, whereas others said it was merely the first job they found upon arriving in the U.S.

#### Job-Related Knowledge

Due to varying tenure in the dairy industry, some participants had much more job-related knowledge than others. Nonetheless, participants demonstrated serious gaps in knowledge in the areas of animal health and especially animal behavior, as well as human–animal interactions. Participants generally understood the relationship between careful observation, feeding, cleanliness and appropriate treatment of animals, and greater animal health and milk production. Some participants discussed how animal stress levels can affect their productivity and increase the risk of injuries in both animals and humans. Some common diseases and pathogens were mentioned as well as the possibility that animal diseases can be transmitted to humans through direct contact. When it comes to animal behavior, and more specifically, how to effectively and safely move animals from and to their pens, participants had a general lack of knowledge and reported a number of misconceptions. For instance, some expressed a viewpoint that cows become more obedient over time as they come to know and respect the workers, and some misidentified aggressiveness as playfulness and curiosity as aggression.

#### Work Ethic/Motivation

Many participants reported having a strong work ethic, with some pointing to pay and recognition as the primary drivers motivating them to work hard. A number of participants mentioned worker motivation as a key to achieve maximum performance, for instance, one worker explained:

Of course, being motivated is very important for any person. If we’re motivated we have more happiness, more ways to perform our jobs better. (FG #6)

Suggestions on how to improve worker motivation included recognizing hard work and providing rewards and incentives. Some expressed a desire for increased oversight, so dairy management could stay more informed about who was or was not doing their job well as a way to foster healthy competition. Others suggested holding more meetings between managers and workers to allow more opportunities for workers to express their opinions for how safety and productivity could be improved. For example, one worker recalled having such meetings in the past in which the workers would be rewarded for not making mistakes:

There was a time when those meetings were held when they [dairy managers] would even bring us pizza and all three shifts got together and shared their different opinions about how the parlor was being managed …. And they used to motivate the workers to keep working and to keep good milk quality. (FG #6)

### Work Itself

Overall, the participants described their jobs as having a great deal of challenging manual labor, time pressures, and related stress. Some described their job tasks as routine and repetitious, whereas others indicated it was non-routine. A number of participants expressed a lack of clarity regarding their role and daily responsibilities. While describing their work, participants primarily focused on the workload, shift and work schedule issues, and the hazards encountered while working with animals.

#### Workload

Overall, participants perceived their jobs as comprising the workload of two to three workers and highlighted the crucial role of teamwork in getting everything done. Participants emphasized the negative impact of high workload and pressure to work fast on safety and productivity. For example, one worker exclaimed:

Sometimes one fees a lot of pressure because you have to clean pens, add bedding, do everything in one day. One is so rushed, that we don’t do our job well. We do it well, but not as well as we should, with perfection. We do it rushing. (FG #3)

Workers expressed a desire for a decreased workload and more adequate time to do their jobs well. It was suggested that this would be accomplished either through more clearly delegated responsibilities or hiring additional workers.

#### Shift and Work Schedule

Two dairies had two 12-h work shifts and the third dairy had three 8-h work shifts. At one dairy, workers reported that they were often required to alternate working day and night shifts and emphasized the difficulty in adjusting to different work and sleep schedules. Participants reported having between 1 and 2 days off per week; some expressed frustration that days off were often during the week and not on weekends.

The primary issue in relation to work shifts was the lack of consistent oversight across shifts. Workers expressed frustration that work quality varies across shifts, but managers were not present to observe who was at fault in these situations, so everyone was blamed. Many of the night shift workers reported difficulty getting their needs met (e.g., equipment repairs) due to lack of management presence.

#### Animal Handling Hazards

Participants spoke about the hazards associated with animal handling, often illustrating their points by describing experiences of accidents and near misses. The most common injury mentioned was being kicked, trampled, or crushed by a cow. Participants described some of the strategies utilized to avoid animal handling hazards (e.g., staying quiet around the cows). However, due to the unpredictable nature of the cows, animal handling accidents were often viewed as non-preventable. One worker described animal handling hazards as follows:

We are working with animals and we have to be in constant physical contact with them because I cannot make a cow go in the chute by telling her to get in. She is not going to get in. I have to be physically there with her. And sometimes she gets scared, and have stepped on or kicked me. There is no way to prevent this. (FG #2)

Getting more assistance from other workers was suggested as a way to help meet some of the demands inherent in their work and overcome animal handling hazards.

### Physical Environment

Participants reported numerous environmental hazards on the dairy, including those related to electricity, unsafe conditions (e.g., wet floors, insufficient light, loose stairs), and exposure to chemicals, dust, manure, contaminated water, and other harmful substances. Sometimes, hazards were mentioned in relation to injuries or illnesses that had occurred as a result (of which some caused missed work days), and sometimes they were mentioned out of concern that they could pose a risk to human and/or animal health and safety. Overall, participants felt environmental hazards were not addressed in a timely fashion. Instances in which hazards were not attended to until multiple workers had been injured were also reported.

The milking parlor was mentioned as an especially high-risk area of the dairy, both due to the aforementioned environmental hazards and animal handling hazards. Participants stressed the importance of addressing environmental hazards more quickly and providing job-specific training on environmental hazards. The use of security camera footage was suggested as a useful way to identify environmental hazards that need to be addressed.

### Equipment

Participants brought up a number of equipment-related factors that influenced safety and productivity outcomes on the dairy. These factors fell under three broad categories: machinery hazards, resource management issues, and personal protective equipment (PPE).

#### Machinery Hazards

Various hazards related to operating milking equipment, tractors, and other heavy machinery were reported. Some of these hazards were related to the dangerous nature of the machinery, whereas others were due to insufficient upkeep and maintenance. Some expressed an opinion that dairy managers should be responsible for maintaining equipment. For instance:

In the milk machines there are a lot of issues that can cause accidents … [it] is not so much the worker’s fault, but that the owner should be responsible of knowing that the machines are in good shape. (FG #6)

In addition to emphasizing more frequent maintenance, participants suggested the importance of job-specific training regarding correct and safe usage of hazardous machinery.

#### Resource Management Issues

Participants mentioned the negative impact of inadequate equipment maintenance on milk quality and their ability to be productive. Instances in which operations had to stop and when insufficient maintenance on one piece of machinery led to breakdowns of other machinery were also cited. Many participants, especially those working in the night shift, complained that they were unable to get equipment repaired in a timely fashion, either because they lacked the necessary training to do so themselves and were unable to access maintenance personnel or because they knew how to fix the problem but were not given permission or the necessary tools to do so. In these situations, some workers attempted to repair broken equipment themselves (even if against the rules), whereas others were afraid to try. In general, participants reported a great deal of resourcefulness and innovation in dealing with these issues. For instance, one worker explained:

[We] have tools, but they’re not the right tools for the job, so we have to look for something, to be creative with new ideas on how to solve problems. (FG #4)

Participants also reported inaction on behalf of management related to broken equipment and feared blame and angry reactions by managers when reporting broken machinery. For example, one worker described a situation in which he reported broken equipment to his manager as follows:

I was told they would fix it right away, and nothing happened. Then the next day I would remind them to fix it and they would say as an excuse that they had forgotten about the [equipment], and that it would get fixed right away, again, I waited that day and until the next afternoon. I waited for an entire week for them to fix the [equipment] and I had to work and clean the parlor, and I ended up all wet. Wet my hands and sleeves. And if I keep getting wet, I mess up my hands then I’m not able to come to work. (FG #4)

Suggestions for how to overcome resource management issues included more frequent maintenance, providing the workers with permission and the necessary tools to fix the equipment themselves, and always having a maintenance person available.

#### Personal Protective Equipment

Participants commented on the availability and use of PPE, such as safety goggles, protective sleeves, gloves, and seat belts. Availability of PPE was varied. Two of the three dairies provided eye goggles and required workers to wear them in certain areas of the dairy or they would risk getting a warning. A number of reasons why workers resisted using PPE were mentioned, including not fully understanding risks, the negative impacts of PPE on their ability to perform their job (e.g., eye goggles fogging up), inconvenience (e.g., seat belts annoying to buckle and unbuckle), and obstinate attitudes. For example, one participant spoke to the discomfort some workers have with PPE due to lack of familiarity:

Sometimes you feel uncomfortable using things that you never used before. For example for us [workers] it’s very odd to work with gloves … at least in our home countries. Here [USA] for a number of things, we use different [safety gear]. (FG #7)

Participants generally recognized the importance of wearing PPE and expressed a desire to have more PPE available to them, specifically citing ear protection for those working in high noise areas, face masks to protect from small particle inhalation, and helmets to protect from cow kicks. Participants emphasized that PPE use should be mandatory in high-risk areas and enforced by dairy management.

### Social–Psychological Environment

With regards to the social–psychological environment of the dairy, participants described their relationships with their fellow coworkers and dairy management, communication barriers and facilitators, and cultural differences that influenced their work relations.

#### Relationships with Coworkers and Dairy Management

Overall, participants described their relationships with their coworkers as positive and supportive, although some described tensions perceived as stemming from poor work performance and irresponsible behavior of others. Participants reported varying quality of relationships with their managers, ranging from mostly positive to mostly negative to non-existent. Workers with positive relationships with their managers emphasized the importance of trust and respect, for instance:

Our supervisors have earned our trust, and here we treat each other like family. As of today, we have never been disrespectful to each other, and that is the most important thing. (FG #2)

Reports of negative interactions with managers, characterized by inaction, dismissiveness, blame, threats, and lack of respect, were common. Overall, participants felt undervalued and some attributed this to discrimination. For instance, one worker stated:

They (bosses/managers) are seeing all the work that we are performing, they have seen good production an all that, good improvements and they don’t value us. It’s the devaluation of the person. The plain fact that we’re Mexican does not mean that we’re something strange. We’re not less than another person. (FG #4)

Workers also commented on the lasting effects of negative treatment by managers:

My supervisor could make threats to me, such as telling me that they’re going to take my [house] away. One carries all those little things here [in my mind] for the rest of your life. (FG #2)

#### Communication

Participants spoke to the important role of communication in terms of promoting both safety and productivity. There were varying perceptions regarding the current state of communication on the dairy; some perceived it as sufficient and others identified room for improvement. The workers generally described within workgroup communication as strong but called for more integrated communication across areas of the dairy. For instance, one worker described communication on the dairy as follows:

Most of the time, no one communicates, no one talks to each other. Each one does their own jobs, and communicate in our tasks, with coworkers in our own areas. But for example, I don’t go tell them ‘what do you think about how I am doing my job?’ I don’t speak with the milkers. (FG #1)

Many participants viewed language as a barrier to communication, describing the English/Spanish divide between workers and managers and the Spanish/Spanish divide among workers from different linguistic backgrounds. For instance, one worker explained the Spanish/Spanish divide as follows:

The problem is sometimes English is not so much the problem, but instead is the diversity of Spanish because they’re from all different parts and use different slangs and vocabulary. There are words I say that mean something different for them. The Spanish language is more complex than the English language (FG #3)

Unsurprisingly, efforts made by dairy management to learn some Spanish and by workers to learn some English were perceived as beneficial to facilitate communication. Participants mentioned the key role of English speaking coworkers as translators in facilitating communication with their bosses. However, participants also noted situations in which translators had misrepresented their words, often to their own benefit, which led to mistrust and frustration. For instance, one worker described having this experience as follows:

If the manager [who speaks Spanish or the interpreter] is angry at you or does not like you, he can do you harm. Sometimes they don’t say what it is (what you tell them) or sometimes they take the credit. What you say to them, a good idea about work, they keep it to themselves and then tell the boss. They keep it and then they don’t speak on your behalf. They tell it so that it favors them. (FG #4)

Others disagreed with the assertion that language was a barrier and perceived other factors to be at the root of failed communication, such as personal issues between workers, managers not listening or paying attention to workers, or managers holding attitudes that they are above the workers. Suggestions to improve communication across the dairy included more frequent meetings, incentivizing cross-area teamwork, utilizing unbiased translators, providing English classes for workers, and creating an environment in which workers both have the opportunity to and feel comfortable speaking up about their needs.

#### Cultural Differences

Participants mentioned a number of perceived cultural differences between American and Latino/a culture and between different groups of Latinos/as. When asked about cultural differences, workers spoke to perceived racism and discrimination both within and outside of the dairy. Workers across all dairies felt that they were mistreated because of their ethnicity, and illustrated this by providing examples of how American workers are given preferential treatment (e.g., given higher pay and easier jobs, allowed to take more breaks), while Latino/a workers are treated as though they can be easily replaced. One worker described this dynamic as follows:

When there’s an accident that we [Latino/a workers] do, they [managers] take [the opportunity] to say ‘Do you want a salary? With those things that you do, with all that you break?’ But if they were American, they [managers] would say, ‘We will immediately fix. It is under warranty.’ They take it to the mechanic. Between them [American workers and managers], there’s a union. Nothing happens. But if it is us … (FG #4)

Overall, participants highlighted important points of intervention to strengthen relations and communication between coworkers and dairy management and to overcome some of challenges stemming from cultural differences.

### Management/Organizational Factors

The primary themes that fell under the category of management/organizational factors were job characteristics, safety policies and procedures, management characteristics, and training (both job and safety).

#### Job Characteristics

When describing the characteristics of their jobs, participants primarily focused on job titles and priorities, work organization, and benefits.

##### Job Titles and Priorities

Participants represented various job titles, including milker, calf caretaker, inseminator, hoof trimmer, corral keeper, and cow pushers (*pushadores*). Some described themselves as wildcards who were trained in all jobs and could fill in for absent workers. Overall, participants suggested a high level of lateral mobility across positions, primarily driven by high turnover and need rather than worker preferences or choice, and low levels of upward job mobility. Some believed upward job mobility was limited due to ethnicity, for example, one worker commented:

Maybe there are people [immigrant workers] capable of becoming bosses, but the “patron” (the boss) will not accept a person that is not from here [U.S.]. (FG #6)

There was variation in tasks and responsibilities reported by workers holding the same job titles across the three dairies, suggesting the importance of training new workers even if they had previously held the same title at another dairy. When asked about their job priorities, participants emphasized the importance of cow health (e.g., making sure cows/calves are eating, detecting and treating sick cows, keeping corrals clean so cows do not get infected, understanding and treating cows well). Participants also mentioned the importance of personal safety and recognized the link between safety, performance, and success of the company. For example, one worker explained:

Safety in a business, regardless of the size is very important, number one. Because safety goes hand in hand with production. If a company has low number of accidents, then it would receive more investments than a company that has too many and high amount of accidents. (FG #2)

When asked about job priorities, participants also stressed the importance of paying attention and being alert, following the rules, acting responsibly, and having good communication.

##### Work Organization

On the whole, participants felt that there was room for improvement in terms of the organization of their work. Although high levels of teamwork were reported within work groups, the overall organization of the dairies was described as siloed. Some suggested the importance of more integrated collaboration and frequent communication across different areas of the dairy to improve efficiency. For instance, one worker described:

We’re working in the same company, however, the workers at the milking parlor do their job, the calf feeders do their job, the outside people do their job. For example, I have had the opportunity to work in the milking parlor, and there are times that when they’re behind, like we get behind. There’s a lot of people there, but they don’t lend a hand, don’t help. It is like that. I’ve seen that it is the same in the other area …. I think it is lack of organization. (FG #1)

One dairy held monthly meetings to discuss safety and productivity, which facilitated cross-area communication and understanding; yet, the workers at this dairy still felt there was room for improvement. Suggestions for improving work organization included maximizing the fit and integration of jobs across the dairy and hiring more employees, so the same worker does not have to do multiple jobs at once.

##### Benefits

All dairies offered room and board to their workers; a number of additional benefits were provided across the dairies (e.g., English classes, vacation time, dental plans). Participants expressed frustration with low pay, lack of overtime pay, and inability to get a raise. For instance, one worker exclaimed:

It’s not good when the boss observes that you’re a good worker and the years go by and there’s no salary increase and it’s always for the same amount of money. (FG #5)

Some Mexican participants reported an inability to advocate for pay raises after the dairy started employing Central American workers. For instance, once worker explained:

If you go to the boss and ask for a salary increase, he would replace you for one of these [South American] workers. (FG #5)

Participants reported a lack of knowledge about health insurance; some were unaware of their coverage status, while others reported insufficient knowledge regarding the specifics of their coverage. Workers reported instances in which they had been led to believe that injuries and illnesses that occurred as a result of work would be covered, only later to find out that they would be financially responsible for all health-care costs.

#### Safety Policies and Procedures

Overall, participants reported limited knowledge regarding the safety policies and procedures of their dairy. It was unclear if this was due to a lack of policies or procedures or lack of awareness on behalf of the workers. Some participants stated that they were required to report incidents, but others were unsure of what to do in the event of an accident. On one of the dairies, the workers were required to talk to the owner before seeking treatment for illnesses or injuries, posing a particular problem for the night shift workers (i.e., because the owner was not available). One worker felt the managers did not believe workers when they reported accidents:

The same applies for when you have an accident, that the supervisors don’t believe you. When we have an accident, you go to the doctor, and they [managers] believe once they see the medical report of the accident. (FG #3)

When discussing safety policies and procedures, participants from one dairy suggested instituting regular doctor’s exams and vaccinations (e.g., tetanus, flu, rabies) for all workers.

#### Management Characteristics

In addition to the dairy owners, the three dairies had a middle layer of managers/supervisors who dealt directly with the workers regarding day-to-day operations. Many workers held negative opinions of dairy management related to a lack of sufficient training, prioritizing cost cutting over worker well-being, taking credit for worker accomplishments, not seeing things from the workers’ point of view, and failing to follow through. Participants perceived dairy management as prioritizing cow health over worker health, mentioning lack of first aid supplies as a way to highlight this point. For instance, when talking about an instance when a worker was kicked in the face by a cow, one worker described the managers’ reactions as follows:

Instead of seeing or worrying about a worker’s face, they’re looking at the cow’s legs to make sure they are not hurt. (FG #4)

Many participants reported a lack of job control and limited ability to contradict their supervisors, even if they felt they were in the right. For instance, one worker described this as follows:

You can’t contradict the bosses. For them, what they do is always the best, even though we can tell them we know a better and more efficient way of doing the same thing. It’s always what they say at the end. (FG #4)

Accessibility of managers was perceived as important to the workers’ ability to successfully do their jobs, particularly in the event that something breaks down. Overall, managers were reported as being less accessible during night shifts.

Participants expressed a number of desired management characteristics, such as being available for communication, understanding of workers and company politics, fair and respectful when reprehending employees (rather than placing blame and getting angry), and well trained (in terms of the work itself and management). For example, one worker described the importance of having a well-trained manager as follows:

It’s very good when you have a supervisor, to have a supervisor that knows, that understands the job. Not to have a supervisor that comes to give orders and tell everybody how to do their jobs, without him really knowing how to do the job. (FG #1)

Other desired characteristics included continuously teaching and training workers, recognizing and appreciating hard work, working side by side with workers, maintaining a relationship based on trust and mutual learning with workers, following through on worker requests in a timely fashion, clearly delegating tasks and responsibilities, holding regular meetings with workers, listening to workers and giving them opportunities to demonstrate new ways of doing things, and providing enough oversight to ensure accountability and quality without micromanaging.

#### Training

Participants discussed the accessibility, content, frequency, and quality of training, both in terms of job/task training and safety training.

##### Job/Task Training

When asked about job and task training, the most commonly mentioned format consisted of on-the-job training provided by a coworker or superior; outside instructors and videos were also mentioned. Overall, participants perceived on-the-job training from an experienced coworker or manager as more valuable than training through a course or video. For instance, one worker commented on his preference for on-the-job training as follows:

It’s better to have a person [with experience] to teach step by step everything, with gestures, with his voice …. If you’re watching a training video, and you get distracted for a while, then you missed a step you were supposed to do. You learn better if someone is there to teach you along the way. (FG #6)

The extent of training received varies across workers – some felt that they had received sufficient training, while some reported receiving no training at all. One worker explained situational factors that contributed to whether or not a new worker received job training:

If the dairy is full, then they have the three milkers, and the boss (owner) is present, you’ll kindly get trained. However, if you get started when someone is missing, they’ll briefly tell you ‘You need to do things this and that way’ and you’ll have to get started at that moment, and you’re told to do it alone. (FG #6)

With regards to training content, workers noted a difference between training content and reality of the day-to-day job. The importance of making sure the individuals doing the training are experienced, educated, good at teaching, friendly, and considerate of the worker was emphasized. Overall, workers expressed a desire to receive additional training, especially focusing on tips to improve task effectiveness and efficiency and explanations for why certain things should be done in certain ways.

##### Safety Training

Overall, participants perceived safety training as important and valuable. Some reported receiving monthly safety trainings, whereas others reported receiving no safety training at all. For instance, one participant explained:

Here [at this dairy] I have only had 3 jobs. And here I have never been told what risks are involved with the job, or what type of accidents I could suffer. Nothing. (FG #6)

Although some participants stressed the importance of safety training for newer, less experienced workers, others felt that it was necessary for safety training to be ongoing as a way to remind even the more experienced workers of how to stay safe on the job. For instance, when asked about safety training, one worker explained:

They’re important and it is good because this way one can also be reminded about the accidents that happen, and avoid committing the same mistake that one sees in the videos, what someone did wrong and how it got hurt. (FG #7)

Various safety training formats were mentioned, including safety meetings, formal courses, videos and written materials, and informal training from coworkers. Participants also mentioned learning about safety through accidents and near misses and through previous jobs. In-person and video-based safety trainings were generally perceived as more beneficial compared to written formats. For example, when asked about video versus written training materials, one worker commented:

[Video format] is better because with the video you get to watch and not read it. Maybe you read it and you don’t understand it. By watching, you get a clearer idea. (FG #3)

Some participants complained that the safety training received was not specific to dairy work, but rather focused on general safety issues (e.g., electrical safety, CPR, weather issues, first aid) or other issues not related to dairy work. For instance, one worker exclaimed:

The safety training videos teach us how to lift boxes, but here at the dairy we don’t lift heavy boxes. It’s rare the time we have to do such a thing. (FG #2)

Some participants suggested that safety training is necessary but not sufficient to protect worker health, stressing the importance of workers taking responsibility for their own and others’ safety as well as the role of machinery maintenance and upkeep.

## Discussion

The findings from this study provide important insights into the experiences of immigrant Latino/a dairy workers, with a focus on the factors that influence health and safety outcomes. The participants identified numerous individual, organizational, environmental, and social–psychological points of intervention for better managing, training, and creating a more safe and productive environment for immigrant dairy workers. Many of the factors identified (e.g., job control, psychological demands) fall under the National Occupational Research Agenda’s organization of work framework, which refers to a range of organizational practices related to production, management, and the ways in which jobs are designed and performed (28). There is substantial research linking organization of work components with various worker health and safety outcomes [e.g., Ref. (29–32)].

Overall, participants advocated for enhanced cross-area integration and a greater voice given to workers. Workload and shift/scheduling issues were identified as particularly stress-inducing job characteristics; efforts should be made to reduce these stressors. Participants emphasized the need to more quickly address environmental hazards and equipment issues during all shifts in order to prevent risks to animal and human health and to optimize productivity. By promptly addressing workers’ concerns and having protocols for communicating when problems arise, managers could reduce issues with productivity and alleviate workers’ frustration.

Of concern, participants demonstrated serious gaps in knowledge in the areas of animal health, animal behavior, and human–animal interactions. Participants also reported limited awareness of transmission of zoonotic diseases. Farm animals are an important source of diseases to humans through direct contact with animals, their environment, or ingestion of contaminated food (33, 34), and agricultural workers in frequent contact with animals are at high risk of zoonotic diseases (35). Culturally sensitive training interventions that focus on increasing awareness and modifying behaviors to reduce exposure to zoonotic risks are essential. One of such programs is currently being evaluated for effectiveness by the authors.

Many participants shared a common belief that animal health and safety was prioritized over worker health and safety, a perception found among other immigrant dairy workers in the U.S. (18). It is essential that dairy management develop and communicate comprehensive safety policies and procedures to create a strong safety culture and make workers feel as though their safety is considered as important and critical to the success of the dairy (as much, if not more so, than animal safety). Many participants requested additional PPE that should be made available and required for use, particularly in high-risk areas of the dairy. Additional PPE should be introduced with training regarding its importance and proper use.

In terms of the social–psychological environment, participants identified a number of strategies to overcome communication barriers, including the use of unbiased interpreters, holding more frequent meetings, and creating an environment that promotes frequent and transparent communication across all levels and areas of the dairy. Cultural stereotypes and perceived discrimination surfaced as prominent aspects of the social–psychological environment, suggesting the importance of clearing up negative misperceptions and making a concerted effort to reduce unfair treatment based on ethnicity. These issues are especially important to address given that perceptions of discrimination have been linked positively with work tension (36, 37) and intentions to quit (38, 39), and negatively with job satisfaction (36, 37, 40) and organizational commitment (36, 38). In addition, other cultural factors are important to consider such as the concept of family and its impact in setting priorities, the notion of respect of authority, the idea of teamwork, and the perceptions of health related to the work tasks. Social class, level of education, and immigration status are also relevant aspects to take into consideration.

These results have a number of important implications for dairy management. Participants’ perspectives on the positive aspects of their jobs can be leveraged to recruit and retain skilled workers, a noted challenge within the dairy industry (41). Many participants indicated poor relations with dairy managers, characterized by low levels of manager accessibility, less than adequate communication, and high levels of management mistrust and inaction. Marín et al. (42) found similar supervisory practices toward Latino/a immigrant workers in poultry processing plants in North Carolina. It appears that at least some of the managers from the dairies involved in this study would benefit from participation in management and leadership training programs. Previous research has demonstrated a link between leadership and improved safety climate and reduced injury rates (43–45).

Participants called for enhanced clarity from management regarding benefits (especially health insurance) and role responsibilities. Many participants also identified ways in which they felt had been unfairly treated, particularly in terms of job mobility, benefits, and pay. Employee perceptions of fairness are associated with positive outcomes, such as performance, organizational commitment, and job satisfaction, whereas perceptions of injustice are linked with negative outcomes, such as high turnover and counterproductive work behaviors (46, 47). Dairy managers should also focus on enhancing perceived job control, in terms of both task autonomy and employee engagement in decision-making, as perceived control has been associated with high levels of performance and motivation, lower stress, and reduced absenteeism and turnover (48). Participants also expressed a strong desire to receive more recognition and appreciation for their work as a way to improve morale and foster motivation. It is important that workers feel valued and listened to.

The findings from this study also have a number of implications for job and safety training. A majority of participants had no previous dairy experience and many had no experience working with animals, suggesting the importance of ensuring all employees receive adequate job task and safety training. Given the scope of variability in previous work experience, it is important not to assume even basic task and safety knowledge among workers (18). Participants spoke to the need for providing initial training to new employees as well as refresher training to those with more experience, suggesting that training should be approached as an ongoing process rather than a one-time event. Training programs should also recognize and account for the diversity across workers in terms of language, education level, and culture (18). Utilizing various different media (e.g., flip charts, fotonovelas, theater) and formats (e.g., visual, verbal, hands on) can help to accommodate different backgrounds and learning preferences. Participants called for more in-person, on-the-job training delivered by experienced and qualified trainers. There is a large literature base suggesting that trainings based on active participation are more effective than lecture-based trainings (49). Additionally, training should focus on correcting misperceptions regarding the preventability of animal handling accidents. Training content should be carefully tailored to reflect the demands and day-to-day realities of dairy work. There have been recent efforts attempting to design culturally relevant, bilingual safety training based on the attitudes, beliefs and practices of Latino/a dairy workers (50); however, more research is needed to assess the effectiveness of these programs.

There are a number of limitations that affect the generalizability of these findings, including the small sample size and the use of convenience sampling methodology to recruit dairies. Workers from different dairies that would not know each other would have been optimal. This was considered and logistics to find a convenient place for everyone, including travel time, requests for time off, and lodging, were major obstacles. It is also likely that participants held back in their comments out of fear that full disclosure would lead to negative consequences stemming from dynamics between workers and dairy management and/or among coworkers (e.g., angry management, job loss). However, the focus groups were a vehicle to start a conversation initiated with the participant observation where rapport had been established and, as previously mentioned, a number of steps were taken to reduce the workers’ fear of retribution.

Future research should attempt to capture the perspectives of a larger number of dairy workers from a representative sample of U.S. dairies based on geographic location and size. It is also important for future research to gain the perspectives of dairy management in terms of the best ways to enhance safety and productivity.

## Conclusion

With the growing prevalence of immigrant Latinos/as in the U.S. workforce, evidence-based research is needed to help organizations develop culturally congruent policies and practices in accordance with the job-related attitudes, values, and behaviors of these workers (51). Results of this study shed light on dairy workers’ perceptions regarding workplace health and safety risks. Although dairy operations are perceived as risky environments, the role of management was clearly highlighted as pivotal in setting a culture of safety and health. Management’s leadership skills can influence workers’ perceptions dramatically. Practicing timely and clear communication, promptly addressing health and safety concerns, readily supplying necessary tools and PPE, and providing adequate feedback can reduce the frustrations shared by participants and improve motivation among dairy workers. Dairy operations should invest not only in culturally congruent training programs for their workers but also in the development of middle and top managers’ human resource management and leadership skills. Despite the inherent limitations, this study serves as a first step toward understanding immigrant Latino/a dairy worker perspectives related to health and safety.

## Author Contributions

All authors (LM, FP, TT, LS, JR, and NR-M) have (1) contributed substantially to the conception or design of the work and/or the acquisition, analysis, or interpretation of the data for the work, (2) participated in drafting the work or revising it critically for important intellectual content, (3) approved the final version to be published, and (4) agreed to be accountable for all aspects of the work in ensuring that questions related to the accuracy or integrity of any part of the work are appropriately investigated and resolved. LM contributed to the design of the work, analysis and interpretation of the data, and drafting and revising of the manuscript. FP and TT contributed to the design of the work, acquisition, analysis, and interpretation of the data, and the drafting and revising of the manuscript. LS and JR contributed to the design of the work and the drafting and revising of the manuscript. NR contributed to the analysis and interpretation of the data, and the drafting and revising of the manuscript.

## Conflict of Interest Statement

The authors declare that the research was conducted in the absence of any commercial or financial relationships that could be construed as a potential conflict of interest.

## Footnotes

^1^www.wjcny.org

^2^www.ufw.org
